# Why we hug: Individual differences predict emotional touch memories

**DOI:** 10.1371/journal.pone.0359868

**Published:** 2026-10-28

**Authors:** Anna Lena Dueren, Paul M. Jenkinson, Aikaterini Vafeiadou, Athanasios Koukoutsakis, Claudia Hammond, Natalie C. Bowling, Aikaterini Fotopoulou, Michael J. Banissy

**Affiliations:** 1 School of Psychological Science, University of Bristol, Bristol, United Kingdom; 2 Department of Psychiatry and Psychotherapy, University Hospital Bonn, Bonn, Germany; 3 Faculty of Psychology, Counselling and Psychotherapy, The Cairnmillar Institute, Melbourne, Australia; 4 Research Department of Clinical, Educational and Health Psychology, University College London, London, United Kingdom; 5 School of Psychology, University of Sussex, Brighton, United Kingdom; 6 School of Human Sciences, Institute for Lifecourse Development, University of Greenwich, London, United Kingdom; Nanjing University, CHINA

## Abstract

Hugging is a ubiquitous form of social touch, yet little is known about how individual differences shape the remembrance of such experiences. In a large-scale survey (*N* = 15,049), we investigated whether various traits predict the likelihood of recalling a hug for its positive emotional impact and the reasons why it was remembered. Participants were recruited through promotion on public broadcasting programs (BBC) and other media. Overall, 69.5% of participants reported recalling a hug for its positive emotional impact. In regression analyses, empathy, body perception, and attachment-related anxiety were associated with a higher likelihood of such recall, whereas attachment-related avoidance was associated with a lower likelihood. Men were more likely than women to report physically arousing hugs as the most memorable. These findings suggest that hugs are predominantly remembered because they convey comfort, support, and understanding, and that individual differences shape autobiographical memories of hugging.

## Introduction

Affection Exchange Theory (AET) postulates that humans have an innate need to share affection with others and that there are individual differences in how people like to show and receive affection [1]. One common type of affective full body touch is hugging. Hugs can convey a range of meanings, such as greeting or comforting someone [2] and they occur in a range of settings [2–4]. Furthermore, hugging has been associated with positive outcomes on stress measures and improved daily mood [5–12]. Previous affective touch literature has already proposed several pathways by which social touch benefits health, including improved stress coping, modulation of stress‑hormonal and cardiovascular systems, and oxytocin‑mediated bonding and emotion regulation [10,13–15]. Accordingly, current models suggest that affective touch experiences throughout life are related to mental and physical health [14]. Additionally, Fairhurst, McGlone and Croy propose a hybrid communication model of affective touch in which a sender’s affective intent is conveyed through touch to a receiver, whose perception is shaped by bottom-up sensory input, top-down contextual and multisensory factors, and reciprocal feedback between both individuals [16]. These models imply that clarifying the meanings of hugs may be helpful for understanding their health‑related impact. This is especially relevant because most mechanistic work so far has focused on generic affective or social touch (e.g., stroking, cuddling), whereas the specific emotional qualities and appraisals of hugging episodes have rarely been examined explicitly [10,13–15]. Here, we investigate whether hugs are generally remembered as emotionally salient experiences and whether individual differences are related to the emotions associated with hugging. Based on AET, we predict that people generally perceive hugs as emotionally meaningful because they are a way of exchanging affection. Moreover, we predict that individual differences in the specific emotions elicited by hugging are related to psychological traits and experiences, and demographic factors, that have been related to affective touch perception previously (outlined below). We use a UK survey sample to address these questions and specify the investigated individual differences below.

One factor that has been researched heavily in relation to affective touch is gender [17–20]. Regarding hugs, men have shown different lateralisation and arm crossing patterns when hugging other men compared to women [3,21,22]. Furthermore, women were found to experience a stress buffering effect of hugs that was absent in men [12]. Regarding the experience of hugs, it is not clear whether there are gender differences; however, men have previously been found to interpret other people’s behaviours more sexually than women [23]. Here, we address the question of whether men and women differ in the reasons why they remember past hugs.

In addition to gender, previous experiences of touch can influence how touch is evaluated [24–27]. For instance, childhood touch experiences can influence the perception of touch and touch behaviours in adulthood [24–26]. Such research has been conducted primarily with regard to the long-lasting effects of childhood sexual abuse [24,25]. However, it is unclear whether less extreme and negative childhood touch experiences also influence adult perception of hugs. In adulthood, it has been reported that the perception of slow, caressing stroking (activating “c-tactile” mechanoreceptors) differs between people: Individuals who experience affective touch more frequently in everyday life were found to evaluate slow stroking as more pleasant than individuals who experience affective touch less often [27,28]. Yet, whether the quantity and quality of hugging experiences in everyday life influence the emotional value placed on hugs remains unclear.

Another topic that has been researched increasingly in relation to affective touch, but rarely in relation to thoughts about hugging, are attachment dimensions. Attachment describes how individuals relate to other people in close relationships [29]. Insecure attachment can be specified into avoidant or anxious attachment dimensions, with people scoring high in attachment avoidance often displaying a high level of independence and self-sufficiency in interpersonal relationships [30]. Individuals scoring high in attachment anxiety, on the other hand, tend to experience distress when facing separation [30]. People scoring higher on measures of attachment avoidance often dislike affective touch such as slow, caressing stroking or cuddling [15,31–33], whereas the opposite seems to be true for people scoring high in attachment anxiety [26,32,34,35] however, also see the conflicting findings by [15,31,36]. We do not currently know how attachment dimensions influence views on hugging, but based on the work on other forms of affective touch, it seems likely that attachment dimensions also relate to the perception and interpretation of hugs. One proposed pathway is that childhood touch experiences impact attachment dimension, which in turn alters the perceived pleasantness and emotional salience of affective touch [33,37].

An additional trait that may relate to attitudes towards hugging is empathy. One use of the term empathy is to describe a sharing of someone else’s affective state, based on the perception and understanding of the other’s emotion [38,39]. Previous research has linked empathy to both one’s own tactile experience [40] and to the observation of other people’s tactile experiences [41–43]. Individual differences in trait empathy have also been linked to structural brain differences in areas of the brain associated with tactile perception, including the somatosensory and insular cortex [44,45]. These studies suggest that there may be a relationship between individual differences in trait empathy and tactile processing, but the relationship between empathy and hugging has to our knowledge, not been investigated.

Another trait that has been researched in relation to affective touch perception is body image. Previous research has found that individuals with pathological body image perceive affective touch differently from controls. For example, patients suffering from anorexia nervosa perceive (c-tactile) affective touch as less pleasant than healthy controls [46–48]. A review of relationships between eating disorders and sexual behaviours noted that anorexia nervosa and bulimia nervosa have also been related to lower sexual desire and higher sexual dysfunction compared to healthy controls [49]. However, evidence regarding affective touch perception and body image in nonclinical populations is scarce. One study of healthy participants found that individuals who are less satisfied with their body experience a greater longing for hugs, which the authors interpret as nurturing touch [50]. Thus, for healthy participants there is some evidence suggesting that the meaning of affective touch might relate to body image; however, it is unclear whether body image in healthy people relates to the likelihood of remembering an emotionally meaningful hug.

Here, we sought to assess whether participants experienced an emotionally meaningful hug in their lives and, if so, why they remember it. Furthermore, we analysed how individual differences in attachment dimensions, self-rated trait empathy, body acceptance and body perception, and individual differences in opinions about hugs relate to these questions in a large healthy adult sample from the UK (> 15,000 respondents). To this end, we conducted a pre-registered secondary data analysis on cross-sectional questionnaire data drawn from a dataset that explored attitudes and experiences towards touch (The Touch Test – a public science project run in collaboration between our laboratories, the Wellcome Collection, and BBC Radio 4). Within this survey, participants were asked if there was a hug that they remembered because it had a positive emotional effect on them, and if so, why the hug was remembered. Based on the literature introduced above, we predicted that:

- *Hypothesis 1.1*: Most respondents remember a hug that had a positive effect on them. This was operationalized by testing whether the proportion of respondents who remembered a hug for its positive emotional effect differed from what would be expected if remembering and not remembering were equally likely.
- *Hypothesis 1.2*: Hugs are generally remembered for their comforting, supporting, and understanding effects rather than for arousing effects. This was operationalized by testing whether the frequencies of the different reasons given for remembering a hug (comfort, support, arousal, friendliness, happiness, lack of touch, understanding) differed from what would be expected if all reasons were equally likely to be selected. *Hypothesis 1.3*: Men will be more likely to remember hugs for their arousing effects than women. This was operationalized by testing whether the proportion of hugs remembered for their arousing effect differed between men and women beyond what would be expected if reason and gender were unrelated.
- *Hypothesis 2*: Individuals who report more tactile affection during childhood bed routine will be more likely to remember hugs for their positive emotional effect than respondents reporting less childhood bed routine. This was operationalized by testing whether higher childhood bed routine scores were associated with increased odds of remembering a hug for its positive emotional effect.
- *Hypothesis 3*: Individuals who experience more frequent and more pleasant everyday hugging will be more likely to remember a hug that had a positive emotional effect relative to respondents who experience less frequent and less pleasant everyday hugging.
- *Hypothesis 4.1*: Respondents with higher levels of empathy will be more likely to remember a hug that had a positive emotional effect than respondents with lower empathy.
- *Hypothesis 4.2*: Respondents with greater scores on avoidant attachment will be less likely to remember a hug for its positive emotional effects relative to respondents with smaller scores on avoidant attachment, the opposite pattern is expected for anxious attachment scores.
- *Hypothesis 5.1*: Individual differences in body acceptance and/or body perception will not predict likelihood of remembering a hug.
- *Hypothesis 5.2*: Respondents with low body acceptance will be more likely to remember hugs for their comforting, supporting, and understanding effects relative to respondents with high body acceptance. This was operationalized by testing whether lower body acceptance scores predicted an increased likelihood of selecting comfort, support, or understanding as the reason for remembering a hug.

## Participants and methods

###  Participants

Participants were recruited through promotion on public broadcasting programs (BBC) and other media. To participate, respondents had to be at least 18 years old and to have internet access. As per pre-registration, the analysis was restricted to healthy participants by excluding data of respondents who reported current disabilities, long-term condition(s), or impairment(s). Furthermore, in accordance with the pre-registered analysis plan, only male and female UK adults were included in this analysis. The reason for this was that participants from the UK constituted the largest group of participants within the sample, with most other countries not having sufficient sample sizes for individual country analyses. The final sample analysed for the current report consisted of 15,049 healthy adults from the UK. Participants were aged 18–92 years (see Table 1 for participant characteristics). Note that the sample number varies depending on the specific analyses, due to participant dropout and unanswered items (*N* per analysis is reported in the results section).

**Table 1:** Participant characteristics.

Variable	N	Mean	SD
Female (%)	11,343 (75.4)		
Male (%)	3,706 (24.6)		
Age	15,030	56.13	13.80
End date	15,049	17.24	18.67
Childhood bed routine	12,280	3.91	1.26
Difficulty hugging	15,040	2.39	1.02
Disliking hugging	15,040	1.99	1.01
Hugging frequency	11,280	3.57	1.60
Greeting by hugging	12,282	3.91	1.26
Empathy cognitive	12,068	5.91	2.60
Empathy emotional	12,070	5.81	2.25
Attachment anxiety	12,115	20.88	8.31
Attachment avoidance	12,107	16.94	7.32
Body acceptance	12,085	16.67	4.32
Body perception	12,034	225.68	107.71

### Design

Data for this study were drawn from the Touch Test, a survey that explored various aspects of touch attitudes and behaviours in a sample of more than 40,000 respondents. Data for the Touch Test were collected between 21/01/2020 and 31/03/2020. Participation was voluntary, and participants were not compensated for their participation. The study was conducted online, and after starting the survey, participants had seven days to complete the questionnaire. Participants gave written informed consent before completing the questionnaire, which was approved in writing by the Research Ethics & Integrity Sub-Committee (REISC) of Goldsmiths, University of London (project reference 1521).

### Measures

The extracted variables from the Touch Test are presented below. See here for the complete Touch Test questionnaire. The following demographic variables were analysed: age, gender, and date of survey completion (hereafter referred to as ‘end date’). End date was included as a covariate in the present study because data collection fell within the expansion of the COVID-19 pandemic in the UK, which might have impacted touch behaviour [51,52].

The following variables were collected:

*Remembering a hug*: “Is there a hug that you remember because of its positive emotional effect on you?”, answered with yes/no. If participants answered ‘yes’, they were asked for *reasons for remembering a hug*: “Please pick the most important phrase that best describes why”. Respondents could select one option from: 1) Comfort, 2) Support, 3) Arousal, 4) Friendliness, 5) Happiness, 6) It followed lack of touch, 7) It conveyed understanding, 8) Other (followed by a text box where the participant could type their own answer).

*Attachment anxiety and avoidance* were measured by a 12-item version of the Experiences in Close Relationships Questionnaire (ECR-12) [53]. Two subscales measure anxious and avoidant attachment dimensions, with greater scores indicating greater attachment anxiety or avoidance. Response options were measured on a scale ranging from 1 (strongly disagree) to 7 (strongly agree). The subscales were found to have good internal consistencies (α between  .74 and  .87 for both subscales) and adequate convergent validity as assessed by the relationship between ECR-12 scores and psychological distress and relationship satisfaction scales [53].

*Body acceptance* was assessed using a composite score derived from averaging five items belonging to the acceptance subscale of the Dresden Body Image Questionnaire (DBIQ) [54,55]. Agreement with items was scored on a scale ranging from 1 (not at all) to 5 (fully); a higher score indicates greater body acceptance. Cronbach’s alpha of the five items was calculated for the current sample and showed excellent internal consistency with α = .92.

*Body perception:* In addition to body acceptance, we were interested in how the perception of one’s own body relates to touch. To investigate this, a new body perception scale was constructed. Five items were scored on a 0 (never) to 100 (always) slider. The items were: 1) I choose clothes not just because of how they look but also how they feel on your skin. 2) How I think about my body’s attractiveness relates to touch. 3) I think that intimate others will find my body pleasurable to touch. 4) I feel that other people’s physical attractiveness depends on how pleasant it is to touch them. 5) My confidence in my own body depends on how positively I am touched by others. These items were summed to create a body perception score, which had a Cronbach’s alpha of  .72. A one-factor confirmatory factor analysis (CFA) was conducted to examine the proposed structure of the five-item Body Perception measure. The model was estimated using robust maximum likelihood (MLR), with full-information maximum likelihood used to accommodate missing item-level data (*N* = 12,072). All five items loaded positively on the latent Body Perception factor, with standardised factor loadings ranging from  .44 to  .72. Model fit was mixed, robust χ^2^(5) = 616.87, *p* < .001, CFI = .94, TLI = .88, RMSEA = .11, 95% CI [.10,  .12], and SRMR = .04. Thus, although the items showed meaningful loadings on a common factor, the overall fit indices did not provide unequivocal support for a strictly unidimensional structure. *Childhood bed routine* was assessed by one item from the Touch Experiences and Attitudes Questionnaire (TEAQ) [19]: “As a child my parents would tuck me up in bed every night and give me a hug and a kiss goodnight”. Scored on a scale ranging from 1 (disagree strongly) to 5 (agree strongly). This item is not exclusively focused on hugs experienced in childhood, but it was here used as a proxy for childhood hugging experiences.

*Difficulty hugging* was assessed by one item from the Glasgow Sensory Questionnaire (GSQ) [56,57]: “I find it hard to know how firmly to hug people.” The item was scored on a scale ranging from 1 (strongly disagree) to 5 (strongly agree), so higher scores indicate greater difficulty hugging.

*Disliking hugging* was also assessed by an item from the GSQ: “I dislike the physical sensation that I get when people hug me.” Higher scores indicate a stronger dislike of hugs.

*Empathy Quotient – Short Form* (EQ-10) [58,59], a 10-item questionnaire designed to measure the ability to empathize in adults. A three-point scoring system was adopted, following previous EQ conventions [58,59]. Forward items were therefore scored 2 for strongly agree, 1 for agree, and 0 for slightly disagree or strongly disagree. Reverse items were scored 2 for strongly disagree, 1 for disagree, and 0 for slightly agree or strongly agree. Items from the emotional reactivity and cognitive empathy subscales were used, with greater scores indicating higher emotional and cognitive empathy. In the present sample calculation of Cronbach’s alpha resulted in α = .59 for the emotional reactivity subscale and α = .84 for the cognitive empathy subscale.

*Hugging frequency* was assessed by the TEAQ [19] item “Most days I get a hug or a kiss”, scored on a scale ranging from 1 (disagree strongly) to 5 (agree strongly).

*Greeting by hugging* was assessed by the TEAQ item “I always greet my friends and family by giving them a hug”, also scored on a scale ranging from 1 (disagree strongly) to 5 (agree strongly).

### Data analysis

Statistical tests were selected based on the nature of the data and the hypotheses under investigation. Chi-square goodness-of-fit and independence tests were used to examine categorical group differences and frequencies, with binomial tests and adjusted residuals providing post-hoc comparisons to identify deviations from expected proportions. Logistic regression models (binomial for binary outcomes, multinomial for categorical outcomes) were employed to predict likelihoods, allowing for the inclusion of multiple continuous and categorical predictors. The specific analyses corresponding to each hypothesis are detailed below.

Hypothesis 1: The frequency of participants who remembered a hug was compared with the frequency of participants who did not remember a hug using a chi-square goodness-of-fit test. Among participants who remembered a hug, reasons for remembering were compared with a chi-square goodness-of-fit test. Participants answering that they remembered a hug for “other” reasons (7.2% of answers) were excluded from analyses for clarity of interpretation. Binomial tests were conducted for post-hoc comparisons. To assess whether men and women remember a hug for similar reasons, a chi-square test of independence was conducted. Adjusted residuals were used for post hoc comparisons, indicating significance when> ± 3 [60].

Hypotheses 2–5: A binomial logistic regression was used to assess whether childhood bed routine, hugging experiences, empathy, attachment anxiety and avoidance, body acceptance, and body perception predicted the likelihood of remembering a hug for its positive emotional effect. To reduce the risk of overinterpreting small effects given the large sample size, the alpha level was set to *p* < .01 with predictors including age, gender, date of completion, hugging quality and quantity in everyday life, and attachment anxiety and avoidance. Odds ratios were used alongside p-values to evaluate the magnitude of effects.

Furthermore, to assess why hugs were remembered, a multinomial logistic regression was conducted with all possible reasons for remembering a hug as the dependent variable. For the multinomial regression, only those individuals were included who ticked “yes” to the question of whether they remember a hug for its positive emotional value. Again, childhood bed routine, hugging experiences, empathy, attachment anxiety and avoidance, body acceptance, body perception, and gender were entered as predictor variables; additionally, age and date of completion were entered as control variables. For brevity, the full model results are reported in the supplemental material.

As pre-registered, for scales and subscales that consisted of several items (body perception scale, DBIQ, ECR-12, and EQ), participants were included if they completed at least 80% of the items of the respective scale. Data were analysed with SPSS 27 (IBM corporation).

### Deviations from pre-registered analysis

Upon inspection of the data, sexual orientation and ethnicity were not included in the subgroup analyses because several categories contained relatively small numbers of participants, limiting our ability to obtain sufficiently precise and interpretable estimates for comparisons across these groups. We therefore chose not to conduct analyses that could produce unstable estimates or encourage overinterpretation of sparsely represented groups.

## Results

Hypothesis 1: What proportion of participants remember a hug, and why? What are gender distributions?

In line with hypothesis 1.1., the chi-square goodness-of-fit test revealed that 10,457 participants indicated that they remembered a hug for its positive emotional value, while 4,589 participants did not remember a hug: a statistically significant difference in proportions; χ^2^(1) = 2288.54, *p* < .001, *N* = 15,046. The chi-square goodness-of-fit comparing reasons for remembering a hug was also statistically significant: χ^2^(6) = 6570.44, *p* < .001, *N* = 9,703, indicating that the different reasons for remembering a hug were not selected equally often, with comfort being the most prevalent and arousal being the least prevalent reason to remember (see Table 2 for distribution). Binomial tests comparing each reason for remembering a hug with all other reasons revealed that all differences were statistically significant; statistics are reported in the Table in S1 Table.

**Table 2:** Reasons for remembering a hug for its positive emotional value by gender.

		Comfort	Support	Arousal	Friendliness	Happiness	It followed lack of touch	It conveyed understanding	Total
Men	Count (%)	556 (25.3)	487 (22.2)	85 (3.9)	270 (12.3)	193 (8.8)	68 (3.1)	538 (24.5)	2,197 (100.0)
	Adjusted Residual	**−8.2**	2.9	**10.3**	**12.4**	1.4	1.2	**−4.9**	
Women	Count (%)	2,598 (34.6)	1,452 (19.3)	62 (0.8)	364 (4.8)	589 (7.8)	196 (2.6)	2,245 (29.9)	7,506 (100.0)
	Adjusted Residual	**8.2**	−2.9	**−10.3**	**−12.4**	−1.4	−1.2	**4.9**	
**Total**	**Count**	**3,154**	**1,939**	**147**	**634**	**782**	**264**	**2,783**	**9,703**

A chi-square test of independence between gender and reasons for remembering a hug was significant; χ^2^(6) = 320.63, *p* < .001, *N* = 9,703, indicating that men and women gave different reasons for remembering a hug. The association was small, with *Cramer’s V* = .18. In line with hypothesis 1.3, adjusted residuals showed that men were more likely than women to remember hugs because they conveyed arousal, friendliness or understanding (Table 2). Women were more likely than men to remember hugs because they conveyed comfort (Table 2).

In total, 754 participants indicated “other” reasons; these participants are excluded here. Adjusted residuals are significant when> ±3.

Hypotheses 2–5: How do hugging experiences in childhood and adulthood, and individual differences in empathy, attachment dimensions, and body representation, relate to hugging memories?

Analysis of studentized residuals revealed 132 potential outliers. Inspection of these cases indicated that they represented genuine observations within the study sample. Given that post hoc exclusion of these cases on statistical grounds alone was considered insufficiently justified and potentially capable of introducing bias, they were retained in the model reported here. Removal of these outliers did not change model statistics significantly, and the Hosmer and Lemeshow goodness-of-fit test showed poor model fit when outliers were removed. Hence, the model reported here includes all data points. The binomial logistic regression model was significant; χ^2^(14) = 1214.97, *p* < .001, *N* = 11,935. The model explained between 9.7% (Cox & Snell *R*^*2*^) and 13.8% (Nagelkerke *R*^*2*^) of variance in the likelihood of remembering a hug and correctly classified 72.6% of cases.

All predictor variables are summarized in Table 3. At *p* < .01, difficulty hugging and disliking hugging were significant negative predictors for the likelihood of remembering a hug that had a positive emotional value. Greeting by hugging, on the contrary, was a significant positive predictor for remembering a hug. Likewise, in accordance with hypothesis 4.1, greater scores on the cognitive and emotional empathy subscales were significant positive predictors for the likelihood of remembering a hug. Attachment anxiety and -avoidance showed contrasting patterns, supporting hypothesis 4.2. Particularly, a higher score on the attachment anxiety subscale was a significant positive predictor for the likelihood of remembering a hug, whereas a higher score on the attachment avoidance subscale was a significant negative predictor for the likelihood of remembering a hug. Participants with higher scores on the body perception scale were more likely to remember a hug that had a positive emotional significance. Effect sizes were small, with odds ratios ranging from 0.871 for disliking hugging to 1.177 for greeting by hugging. Age, gender, end date, childhood bed routine, hugging frequency, and body acceptance were not significant predictors for remembering a hug.

**Table 3:** Binomial logistic regression predicting likelihood of remembering a hug for its positive emotional value.

Predictor variables	OR	95% Confidence Interval for OR		SE	χ^2^	*p*
		Lower	Upper			
Age	1.004	1.001	1.007	0.002	5.71	.017
Gender	0.997	0.901	1.102	0.051	0.01	.946
End date	1.001	0.998	1.003	0.001	0.23	.636
Childhood bed routine	1.018	0.987	1.050	0.016	1.34	.246
Difficulty hugging	0.902	0.861	0.945	0.024	19.10	**< .001**
Disliking hugging	0.871	0.832	0.912	0.023	34.74	**< .001**
Hugging frequency	0.986	0.958	1.015	0.015	0.88	.349
Greeting by hugging	1.177	1.133	1.223	0.020	69.65	**< .001**
Empathy cognitive	1.056	1.037	1.074	0.009	35.89	**< .001**
Empathy emotional	1.079	1.057	1.102	0.011	50.64	**< .001**
Attachment anxiety	1.026	1.020	1.032	0.003	80.46	**< .001**
Attachment avoidance	0.989	0.983	0.995	0.003	11.74	**.001**
Body acceptance	1.002	0.992	1.013	0.005	0.20	.659
Body perception	1.003	1.002	1.003	0.000	165.39	**< .001**

Gender is for men compared to women. OR = odds ratio, SE = standard error.

The multinomial regression with reasons for remembering a hug as the dependent variable was overall statistically significant *χ2*(84) = 679.03, *p* < .001, *N* = 7,763. The model explained between 8.4% (Cox & Snell *R*^*2*^) and 8.7% (Nagelkerke *R*^*2*^) of the variance in reasons for remembering a hug. It correctly classified 35.3% of reasons for remembering a hug overall, with large variance between different answer options (see supplemental material). Significant predictors included age, gender, childhood bed routine and kisses, disliking hugging, hugging frequency, cognitive and emotional empathy, and scores on the body perception scale (Table in S2 Table). Again, most effect sizes were rather small, except the effects of gender on the likelihood of remembering a hug for friendliness (OR = 2.795) and for arousal (OR = 5.960). Hypothesis 5.2 was not supported, as body acceptance score was not a significant predictor for reasons for remembering a hug. However, greater scores on the body perception scale related to a higher likelihood of remembering a hug because it conveyed understanding or arousal, compared to comfort.

## Discussion

Here, we presented an analysis of a large data set investigating the meaning of hugging in adults and how individual differences in a range of traits are related to hugging memories. We found that hugs were remembered primarily because they conveyed comfort, support, or understanding. Individual differences in everyday hugging experiences, cognitive and emotional empathy, attachment dimensions, and body perception were related to the likelihood of remembering a hug for its positive emotional effect. Importantly, the large sample size allowed us to detect subtle but systematic differences in how hugs were remembered, including effects of attachment dimensions and gender that may have remained undetected in smaller samples. These findings suggest that individual differences in attachment dimensions and empathy contribute to which touch experiences are selected as emotionally meaningful and retained in autobiographical memory. This pattern is consistent with hybrid communication accounts of affective touch, in which a receiver’s own characteristics shape the decoding of a tactile signal into an emotional appraisal [16]; our findings suggest that these same receiver characteristics also predict whether that appraisal is subsequently retained in autobiographical memory.

Below, these findings are discussed with respect to each hypothesis.

Hypothesis 1: Do participants remember a hug, and if yes, why?

As expected, most participants remembered a hug that had a positive emotional effect on them, indicating that hugs can constitute an emotionally meaningful stimulus worth remembering. This finding is in line with previous theoretical work [2] and with findings on physiological wellbeing, suggesting that hugs can be a source of socio-emotional support and stress reduction [6,8,11,12,61].

In the current study, the reasons why hugs were remembered were in accordance with data suggesting that hugs provide stress-buffering social support [5,6,8,12,62], as most participants remembered hugs for their comforting, supporting, or understanding effects. These findings suggest that hugs are less often remembered as erotic than other forms of affective touch [63]. Previous quantitative evidence on the association of hugs with social support and stress-buffering outcomes has mostly investigated physiological markers of stress, such as heart rate, blood pressure, stress-related cytokines, and oxytocin levels [5,6,8,12]. In our study, the stress-buffering role of hugs is corroborated by self-reports from a large, diverse adult sample.

Gender was not found to predict the likelihood of remembering a hug. However, we found gender differences in the reasons why hugs were remembered. In accordance with hypothesis 1.3, men were more likely to remember hugs for their arousing effects than women. Furthermore, analysis of adjusted residuals showed that men were more likely than women to remember hugs because they conveyed friendliness, whereas women were more likely than men to remember hugs for their comforting or understanding effects. These findings suggest that the nurturing aspects of hugs may be more important to women than to men, potentially in line with research indicating that men may perceive interpersonal situations as more sexual than women [23,63]. However, fewer men than women participated in the current study and indicated why they remembered hugs (2,197 vs 7,506, respectively: Table 2), therefore, these gender differences should be interpreted with caution. Furthermore, we did not find an association between age and whether participants remembered a hug that had an emotional effect on them [64]. Further studies are necessary to determine the relationship between hugging and age-related changes in affective touch perception.

Hypothesis 2: Childhood bed routine and kisses

Contrary to hypothesis 2, individual differences in childhood bed routine were not found to predict the likelihood of remembering a hug in adulthood. This finding stands in contrast to evidence suggesting that childhood touch experiences are associated with adult touch perception [24–26]. A possible reason for our findings could be that the item used to quantify childhood bed routine (“As a child my parents would tuck me up in bed every night and give me a hug and a kiss goodnight”) is not informative about a participant’s evaluation of the touch described. Moreover, the item conflates multiple behaviours (tucking in, hugging, and kissing), which represents a psychometric limitation and may have reduced its specificity and predictive validity. The evaluation of childhood bed routine might furthermore depend on factors such as whether the touch was abusive or not, and on individual differences in attitudes towards touch. Therefore, the item presented here might not have been sufficient to capture all relevant dimensions of childhood bed routine. Additionally, the accuracy of retrospective measures of childhood experiences has been debated [65]. Hence, the present findings require further investigation and should be regarded as exploratory.

Hypothesis 3: Hugging quality and quantity in everyday life

We evaluated the relationship between hugging preferences, hugging frequency, and the likelihood of remembering a hug (a proxy for whether hugs carry emotional value for a person). It was found that greeting by hugging, difficulty knowing how to hug, and disliking hugging were associated with the likelihood of remembering a hug, whereas frequency of hugging was not. In more detail, difficulty knowing how to hug and disliking hugging were negatively related to remembering a hug for its positive emotional value, while the opposite pattern was observed for individuals who are more likely to greet family and friends by hugging. Our findings suggest that the subjective experience of touch relates to the likelihood of forming a positive memory about it.

Hypothesis 4: Empathy and attachment dimensions

In accordance with hypothesis 4.1, greater scores on cognitive and emotional empathy subscales were related to a higher likelihood of remembering a hug. Previous research found that higher levels of self-rated empathy relate to more positive evaluations of images displaying social touch [41]. The current findings suggest that individual differences in empathy relate to the importance attributed to hugs. Furthermore, it is notable that both the cognitive and emotional subscales of the EQ-10 predicted the likelihood of remembering a hug. The cognitive subscale is thought to describe the ability to understand and predict the feelings of another person [66]. The emotional subscale is considered to describe feelings that are vicariously triggered by witnessing another’s emotional state [66]. The emotional subscale showed comparatively low internal consistency in the present sample, and its contribution to predicting hug memory should therefore be interpreted with caution. Still, differences in emotional empathy have also been linked to tactile perception. One study measured individual differences in empathy and in a tactile discrimination task [40]. It was found that individual differences in sympathy and concern for other people were positively related to performance on the tactile acuity task. The authors discussed the possibility that highly empathic people may have a hyper-sensitive tactile system [40]. Our findings are in line with this. It will be interesting for future work to assess whether hypersensitivity to tactile stimuli in highly empathic individuals is more pronounced for affective than for emotionally neutral touch.

With regards to attachment dimensions, in line with hypothesis 4.2, we found differences in the likelihood of remembering a hug for participants scoring high on anxious attachment and participants scoring high on avoidant attachment. Specifically, participants with greater scores on the avoidant attachment subscale were less likely to remember a hug for its positive emotional value, whereas the opposite was true for individuals scoring higher on the anxious attachment subscale. These findings are in accordance with previous findings on affective touch and attachment dimensions, suggesting that affective touch can attenuate pain in anxiously attached adults [32] and may elicit state attachment security [67]. Further, our findings are in line with work suggesting that greater attachment avoidance is linked to more negative attitudes about affective touch [15,26,31] and that affective touch possesses lower analgesic effects in avoidantly attached individuals [32]. Our findings extend this work to show that individual differences in attachment dimensions are related to individual differences in the emotional importance placed on hugging.

Hypothesis 5: Body acceptance and body perception

Body perception was related to hugging memories: participants with higher scores on the body-perception scale were more likely to remember a hug. In contrast, body acceptance as measured with the DBIQ was not significantly associated with hugging memories. Thus, the findings were only partly consistent with our hypothesis and suggest that touch-related cognitions about the bodily self may be more relevant to hugging memories than general body satisfaction. This interpretation is consistent with the conceptual distinction between the two measures: whereas the body-perception scale focuses on the connection between body awareness and touch, the DBIQ body acceptance subscale assesses how comfortable and satisfied individuals feel in their own body [55]. Accordingly, the present findings indicate that hugging memories may reflect broader touch-related beliefs about the self rather than general body acceptance.

Previous research on the continuity between body acceptance and affective touch perception has been conducted predominantly in clinical samples and found an association between touch perception and body acceptance [46–49,68]. By contrast, the present findings suggest that, in a healthy sample, subclinical variation in body acceptance is not directly associated with whether individuals remember a hug that had a positive emotional impact. It remains unclear whether this pattern generalises to other forms of affective touch or to clinical populations.

Our hypothesis that lower body acceptance would be associated with a greater need for nurturing touch was not supported. Body acceptance did not predict the reasons for remembering a hug (see Table in S2 Table). By contrast, higher body-perception scores were associated with a greater likelihood of remembering a hug because it conveyed arousal or understanding rather than comfort.

### Limitations

The research presented has some limitations. The sample was self-selecting, and respondents did not receive compensation for participation. This may mean that the sample is more representative of people who were interested in the topic of touch. However, it should be noted that the sample analyzed was large and diverse in terms of age (range 18–92 years) and traits measured, hence the statistical power was sufficient to detect even small effects and can be considered representative of a range of individual differences of interest within the study. A further limitation concerns the exclusively UK-based sample. Cultural norms influence how touch is expressed and interpreted [69–71], and the present findings may therefore not generalise to societies with different attitudes towards physical contact and interpersonal distance. Future studies are needed to determine whether similar patterns emerge in non-UK samples. Another topic that needs to be raised is the use of single-item measures instead of validated scales consisting of several items. Single-item measurements have been criticized previously because some psychological constructs might not be captured fully by such measures [72]. However, single-item measures have proved reliable and useful when assessing unidimensional, global constructs [72–74], and most of the constructs we measured with single items are such global constructs. An exception is childhood bed routine, which was assessed by only one item from the childhood touch scale from the TEAQ [19]. Here, the use of this item was exploratory, as the relationship between childhood bed routine and kisses has not been researched in relation to hugging memories before. Therefore, the findings based on this item should be regarded as preliminary. Relatedly, although a confirmatory factor analysis showed that all body perception items loaded meaningfully onto a common factor, fit indices – particularly TLI and RMSEA – did not support a strictly unidimensional structure. Therefore, results including the body perception scale should be regarded as preliminary.

A further limitation is that the data are cross-sectional, which precludes conclusions about causal direction and temporal dynamics between traits, hugging experiences, and the formation of emotionally meaningful hug memories.

Data collection occurred between January and March 2020, with less than 1% of responses collected after 26 March 2020, when enforceable UK restrictions on social contact came into force. The study was thus not designed to capture pandemic-related touch deprivation. Nonetheless, changing patterns of social contact toward the end of data collection may have shaped some participants’ experiences, and touch deprivation, or “touch hunger,” remains an interesting avenue for future research.

Additionally, although the present models were statistically significant, the majority of effect sizes were small and the multinomial model showed only modest classification accuracy. This indicates that the traits included here explain only a limited proportion of the variance in hugging memories. It is therefore likely that additional factors, particularly situational and relational context, contribute substantially to whether and why a hug is remembered. Future studies should also consider broader socio-demographic and family-related variables, such as employment status, living arrangements, and parenthood, which may contribute to everyday touch exposure and the meaning attributed to hugs.

Furthermore, the data did not provide contextual details about the remembered hugs, such as the relationship with the hugging partner or the specific setting, thereby limiting interpretation of factors influencing memorability. This constraint is typical of survey-based designs, where fixed-choice formats reduce data richness. Relatedly, 7.2% (N = 754) of participants selected “other” as their most important reason for remembering a hug. A formal thematic analysis of these free-text responses was beyond the scope of the present study. However, these responses represent a potentially rich source of insight beyond the categories examined here. As the dataset underlying this study has been made openly available, we see this as an opportunity for future research to conduct a qualitative analysis of these free-text responses. Nonetheless, the use of a large and diverse sample enabled the identification of robust patterns across a wide population, providing population-level insights into the role of touch in memory and emotional experience.

Relatedly, the present analyses were primarily descriptive and did not test underlying mechanisms. Future studies could use mediation and moderation models, or person-centred approaches such as latent profile analysis, to clarify how exactly individual differences shape hugging memories.

## Conclusion

While hugging is an important form of everyday touch, there is little research on how and why hugs become memorable. Here, we present the first study to examine autobiographical memories of hugging in a large, healthy adult UK sample. The findings show that hugs are commonly remembered for comfort, support, and understanding. Importantly, individual differences in empathy, attachment dimensions, body perception, and everyday hugging experiences were associated with whether hugs were remembered and why. In this way, the study provides an overview of the psychological factors that shape the meaning of social touch and extends current theoretical work on affective touch by linking affectionate exchange to the formation of autobiographical memories.

## Supporting information

S1 Table Statistics of binomial tests comparing all reasons for remembering a hug in one-to-one comparisons. (XLSX)

S2 Table Multinomial regression predicting reasons for remembering a hug. (XLSX)
